# Association of Dietary Factors with Nephrolithiasis: A Systematic Review and Meta-Analysis

**DOI:** 10.3390/nu18213653

**Published:** 2026-11-03

**Authors:** Nitcha Suphatheerawatr, Rossanun Shoosanglertwijit, Jeerath Phannajit, Kullaya Takkavatakarn, Wanjak Pongsittisak, Aschariya Wipattanakitcharoen, Paweena Susantitaphong

**Affiliations:** 1 Division of Nephrology and Renal Replacement Therapy, Department of Internal Medicine, Faculty of Medicine Vajira Hospital, Navamindradhiraj University, Bangkok 10300, Thailandwanjak@nmu.ac.th (W.P.); 2 Division of Nephrology, Kluaynamthai Hospital, Bangkok 10110, Thailand; 3 Division of Nephrology, Department of Medicine, Faculty of Medicine, Chulalongkorn University and King Chulalongkorn Memorial Hospital, The Thai Red Cross Society, Bangkok 10330, Thailandpesancerinus@hotmail.com (P.S.); 4 Center for Excellence of Metabolic Bone Disease in CKD Patients, Faculty of Medicine, Chulalongkorn University, Bangkok 10330, Thailand; 5 Division of Clinical Epidemiology, Department of Medicine, Faculty of Medicine, Chulalongkorn University and King Chulalongkorn Memorial Hospital, The Thai Red Cross Society, Bangkok 10330, Thailand

**Keywords:** diet, kidney stone, nephrolithiasis, food and drink intake, food, water, dietary intake finding, alcohol intake, nutrient intake

## Abstract

**Background:** Nephrolithiasis is a highly prevalent condition heavily influenced by dietary habits. While individual nutrients have been extensively studied, comprehensive evaluations of broad dietary patterns remain fragmented. This systematic review and meta-analysis aims to quantify the risk of nephrolithiasis across eight distinct nutritional domains. **Methods:** Following PRISMA 2020 guidelines, we systematically searched electronic databases, including PubMed and Scopus (up to May 2026), to identify studies investigating the association between dietary factors and the risk of nephrolithiasis. Multivariable-adjusted hazard ratios (HRs) and 95% confidence intervals (CIs) were extracted. Pooled estimates were calculated using random-effects models, with heterogeneity assessed via the *I^2^* statistic and publication bias evaluated using Egger’s test. **Results:** We synthesized data from 61 unique observational studies. High fluid volume and specific beverages, including coffee, tea, and moderate alcohol, demonstrated strong protective effects. A plant-forward dietary pattern—encompassing high intake of fruits, vegetables, dietary fiber, and adherence to the Dietary Approaches to Stop Hypertension (DASH) diet—was significantly associated with reduced stone risk. Furthermore, dairy products and dietary calcium conferred significant protection, supporting the intestinal oxalate-binding mechanism. Conversely, nephrolithiasis risk was significantly elevated by high consumption of meat, sugar-sweetened beverages, and ultra-processed foods. Metrics of high dietary acid load, specifically net endogenous acid production, strongly correlated with increased risk. Sensitivity analyses restricted exclusively to high-quality prospective cohorts confirmed the robust protective effects of fruit, dairy, coffee, dietary calcium and the DASH diet, alongside the elevated risk associated with sodium and sugar-sweetened beverages. **Conclusions:** Diet plays a profound role in the pathogenesis of nephrolithiasis. Nutritional strategies for stone prevention should transition beyond single-nutrient restrictions toward holistic dietary patterns. A regimen prioritizing adequate hydration, plant-based foods, and dairy, while strictly limiting animal meat, refined sucrose, and systemic acid loads, provides robust protection against stone formation.

## 1. Introduction

Nephrolithiasis, characterized by the presence of crystalline stones (calculi) within the urinary tract, is a highly prevalent urological disorder that imposes a substantial global burden of morbidity and healthcare costs [1]. Its incidence varies significantly by sex, age, race, and geography. According to the Global Burden of Diseases (GBD) 2021 study, nephrolithiasis accounted for approximately 106 million incident cases—of which 67% occurred in men—representing a 26.7% increase globally between 2000 and 2021 [2]. Furthermore, disease-related mortality reached 17,700 deaths in 2021, a 60.3% increase over the same period [2]. Beyond mortality, nephrolithiasis frequently leads to severe clinical complications, including acute renal colic, recurrent infections, obstructive uropathy, and the potential progression to acute or chronic kidney disease, all of which severely strain global healthcare systems.

The etiology of nephrolithiasis is multifactorial, encompassing metabolic syndrome, medication use, lifestyle factors, genetic factors, and, notably, diet. Dietary components directly influence the pathogenesis of stone formation by altering urinary composition. For instance, high dietary protein intake increases acid load and promotes urinary calcium and oxalate excretion [3,4] and elevates uric acid production, particularly purine-rich foods [5]; this, combined with a lowered urinary pH, creates a particularly critical lithogenic condition, while high-sodium diets can induce hypercalciuria [6]. Conversely, the high citrate content found in certain fruit juices is known to increase both urine pH and urinary citrate excretion, thereby exerting a protective effect against stone formation [7].

The association between nutrition and kidney stone formation is well-established, as demonstrated by previous systematic reviews examining specific dietary exposures and combinations of dietary and lifestyle factors [8,9,10]. This systematic review and meta-analysis aims to comprehensively evaluate the associations between all major dietary food groups and the risk of incident and recurrent nephrolithiasis. Our analysis encompasses a broad spectrum of dietary factors—including foods, beverages, specific nutrients, water characteristics, and overall dietary patterns—and provides an updated evaluation of newer exposures, such as ultra-processed foods and dietary acid-load indices. By analyzing this extensive range of dietary exposures, this study seeks to provide a holistic, comparative overview of dietary risk factors to better inform clinical nutritional guidelines for kidney stone prevention.

## 2. Methods

This systematic review and meta-analysis was designed, conducted and reported in accordance with the Preferred Reporting Items for Systematic Reviews and Meta-Analyses (PRISMA) guideline [11]. The PRISMA checklist is available in the Supplementary Materials. The study protocol was registered with PROSPERO (CRD 420261461090).

### 2.1. Search Strategy

We performed a systematic search of electronic databases, including PubMed and Scopus, from inception up to May 2026, to identify studies investigating the association between dietary factors and the risk of nephrolithiasis. The full details of the search strategy are provided in Supplementary Table S1. We also manually screened the reference lists of included articles and relevant reviews to identify additional eligible studies.

### 2.2. Study Selection

Studies were eligible for inclusion if they met the following criteria: (1) included adult participants aged 18 years or older; and (2) reported the risk or incidence of nephrolithiasis as an outcome, providing effect estimates (e.g., hazard ratios (HRs), relative risks (RRs), or odds ratios (ORs)) with corresponding 95% confidence intervals (CIs). Studies were excluded if they met any of the following criteria: (1) involved animal models or in vitro experiments; (2) focused on populations with known genetic conditions predisposing them to nephrolithiasis; (3) exclusively enrolled pregnant or breastfeeding women; (4) were reviews, letters, case reports, editorials, randomized controlled trials, or meta-analyses; or (5) were not available in the English language.

### 2.3. Data Extraction

Data extraction was performed independently by two investigators (NS, RS). Disagreements were resolved through consultation with a third reviewer (PS). The extracted variables included: first author, publication year, study design, total sample size, participant demographics (age and sex distribution), follow-up duration, and dietary assessment tools (e.g., Food Frequency Questionnaires). For exposure factors, we extracted data on all evaluated food categories (such as tubers, vegetables, fruits, beans, dairy, and meat) categorized as binary (high vs. low) or continuous variables. For the primary outcome, we extracted both crude and fully adjusted effect estimates (HRs, RRs, or ORs) with their corresponding 95% CIs. Whenever studies reported multiple statistical models, both crude and the most fully adjusted effect estimates were extracted to account for potential confounding factors. Additionally, we recorded the specific methods used to diagnose nephrolithiasis, such as self-reported clinical diagnosis, medical record or register linkage (ICD-9/10 codes), or objective radiographic confirmation (including non-contrast CT, ultrasound, or KUB X-rays).

### 2.4. Statistical Analysis

All statistical analyses were performed using the Comprehensive Meta-Analysis program (version 4). To evaluate the association between dietary exposures and nephrolithiasis, we calculated pooled effect estimates (HRs, RRs, or ORs) alongside their 95% CIs. Anticipating significant clinical and methodological heterogeneity across the included nutritional epidemiological studies, we synthesized effect sizes utilizing a random-effects model.

Statistical heterogeneity among the studies was evaluated using Cochran’s Q statistic, with *p* < 0.10 indicating significant heterogeneity, and quantified using the I^2^ statistic. I^2^ values of <25%, 25–50%, 50–75%, and >75% were interpreted as representing very low, low, moderate, and high heterogeneity, respectively. Potential publication bias was statistically evaluated using Egger’s linear regression test.

To address the potential for reverse causation inherent in cross-sectional and retrospective designs, we conducted a sensitivity analysis restricted to high-quality prospective cohort studies (assessed as having a ‘Low’ or ‘Some concerns’ risk of bias).

### 2.5. Quality Assessment

The methodological quality and risk of bias of the included studies were assessed independently by two reviewers (NS, RS) using the Risk Of Bias In Non-randomized Studies of Exposures (ROBINS-E) tool. Any discrepancies between the two reviewers were resolved by consultation with a third investigator (PS). Following the domain-by-domain evaluation, each study was assigned an overall risk of bias judgment strictly categorized as one of the following: (1) Low risk of bias, (2) Low risk of bias, except for concerns about uncontrolled confounding, (3) Some concerns, (4) High risk of bias, or (5) Very high risk of bias. Based on the evaluation of these seven domains, the overall risk of bias for each study was categorized as “Low,” “Some concerns,” “High,” or “Very high” [12].

## 3. Results

### 3.1. Study Selection

**Figure 1:**
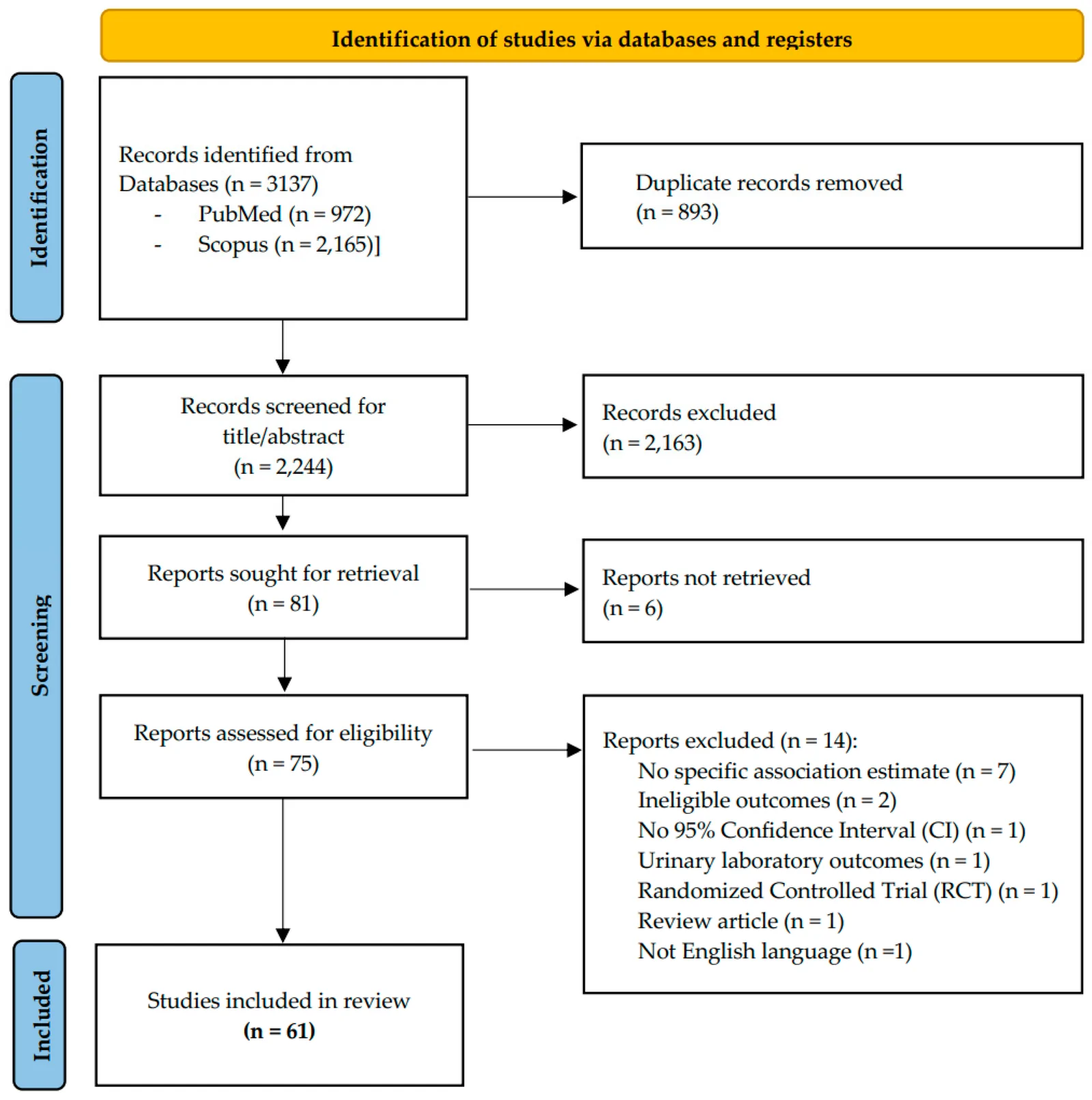
PRISMA flow diagram.

The initial systematic search of the electronic databases identified 3137 records: 972 from PubMed and 2165 from Scopus. After removal of 893 duplicate records, 2244 records remained for title and abstract screening, of which 2163 were excluded as irrelevant to the review objectives. Full texts were sought for the remaining 81 reports, but 6 could not be retrieved. Consequently, 75 full-text reports were assessed for eligibility against the predefined criteria. Of these, 14 reports were excluded for the following reasons: lacked the specified association estimates (*n* = 7), absence of a 95% CI (*n* = 1), assessment of laboratory-based urine profiles rather than clinical nephrolithiasis (*n* = 1), RCT design (*n* = 1), review article (*n* = 1), Chinese-language publication (*n* = 1), and irrelevant outcomes (*n* = 2). Ultimately, 61 studies fulfilled all eligibility criteria and were included in the final systematic review and meta-analysis (Figure 1). The study workflow is shown in Figure S1.

### 3.2. Characteristics of Included Studies

The baseline characteristics of the 61 included studies are summarized in Supplementary Table S2. They were published between 1993 and 2026. The methodological designs primarily consisted of prospective and retrospective cohorts, alongside cross-sectional and case–control studies. Dietary exposures were predominantly captured utilizing self-administered or interview-based validated tools, such as Food Frequency Questionnaires (FFQs) and 24-h dietary recalls. To confirm incident or recurrent nephrolithiasis, the studies employed a variety of outcome assessment methods, ranging from self-reported diagnostic questionnaires to objective confirmation via electronic medical records (ICD codes) and radiographic imaging (e.g., ultrasonography, plain abdominal radiography, and computed tomography). Dietary factors were categorized into eight distinct nutritional domains based on dietary matrix, fluid intake, micronutrient profile, and overall dietary quality (Table 1).

### 3.3. Plant-Based Foods and Dietary Fiber

Six reports evaluated the association between fruit intake and nephrolithiasis risk, whereas three examined vegetable intake. In multivariable-adjusted binary models (highest vs. lowest consumption), significant protective effects were observed for fruits (HR 0.849, 95% CI 0.818–0.881, *p* < 0.001), vegetables (HR 0.910, 95% CI 0.839–0.987, *p* = 0.023), and dietary fiber (HR 0.825, 95% CI 0.766–0.889, *p* < 0.001). Conversely, non-significant associations were observed for leafy vegetables (HR 1.094, 95% CI 0.957–1.250, *p* = 0.190), grains (HR 1.123, 95% CI 0.954–1.322, *p* = 0.163), beans/legumes (HR 1.297, 95% CI 0.869–1.938, *p* = 0.203), tubers (HR 1.190, 95% CI 0.981–1.443, *p* = 0.078), root-stock foods (HR 1.078, 95% CI 0.924–1.258, *p* = 0.340), nuts (HR 1.038, 95% CI 0.886–1.216, *p* = 0.646), and betel nut (HR 1.132, 95% CI 0.994–1.289, *p* = 0.061). Moderate to substantial heterogeneity was observed for dietary fiber (I^2^ = 70.1%, *p* < 0.001), leafy vegetables (I^2^ = 73.6%, *p* < 0.001), and beans/legumes (I^2^ = 73.8%, *p* = 0.001), whereas heterogeneity was low or absent for the remaining plant-based domains (Table 1).

### 3.4. Animal-Based Foods and Protein Sources

In multivariable-adjusted binary models, higher meat intake was associated with a significantly increased risk of nephrolithiasis (HR 1.153, 95% CI 1.100–1.209, *p* < 0.001). Conversely, frequent consumption of dairy products conferred a significant protective effect (HR 0.926, 95% CI 0.886–0.968, *p* = 0.001). Total animal protein (HR 1.006, 95% CI 0.983–1.029, *p* = 0.620), fish (HR 1.020, 95% CI 0.991–1.050, *p* = 0.171), and egg consumption (HR 0.870, 95% CI 0.647–1.170, *p* = 0.356) were not significantly linked to nephrolithiasis risk. Notably, statistical heterogeneity was consistently low to moderate across these animal-based dietary domains. No heterogeneity was observed for meat and fish (I^2^ = 0%), while minor to moderate variance was noted for dairy products (I^2^ = 23.7%), eggs (I^2^ = 33.2%), and total animal protein (I^2^ = 38.6%). These findings were largely consistent in continuous exposure models, where dairy, fish, and total animal protein also yielded non-significant associations with low heterogeneity (Table 1).

### 3.5. Non-Alcoholic Beverages and Juices

Based on six reports, high coffee intake demonstrated a strong protective association against nephrolithiasis (HR 0.845, 95% CI 0.803–0.890, *p* < 0.001), a finding supported by continuous dose–response analyses from three reports (HR 0.909, 95% CI 0.887–0.932, *p* < 0.001). Similar protective effects in binary models were observed for caffeine (2 reports; HR 0.909, 95% CI 0.850–0.971, *p* = 0.005), tea (12 reports; HR 0.925, 95% CI 0.874–0.980, *p* = 0.008), and orange juice (1 report; HR 0.903, 95% CI 0.851–0.958, *p* = 0.001). In contrast, significantly elevated risk was associated with high consumption of sugar-sweetened beverages (2 reports; HR 1.340, 95% CI 1.251–1.436, *p* < 0.001), soda (2 reports; HR 1.067, 95% CI 1.003–1.135, *p* = 0.039), grapefruit juice (3 reports; HR 1.116, 95% CI 1.033–1.207, *p* = 0.006), and punch juice (1 report; HR 1.141, 95% CI 1.067–1.220, *p* < 0.001) (Table 1).

Significant moderate to substantial heterogeneity was observed across several major beverage domains in these binary models, including tea (I^2^ = 70.5%, *p* < 0.001), caffeine (I^2^ = 68.4%, *p* < 0.001), sugar-sweetened beverages (I^2^ = 56.6%, *p* = 0.008), coffee (I^2^ = 54.9%, *p* < 0.001), and soda (I^2^ = 50.9%, *p* = 0.005). Conversely, low or absent heterogeneity was noted for fruit juices, including apple (I^2^ = 17.2%), grapefruit (I^2^ = 11.5%), orange (I^2^ = 0%), and punch juices (I^2^ = 0%) (Table 1).

### 3.6. Alcohol, Water Hydration, and Water Composition

Based on 11 reports, high total fluid and water volume intake demonstrated a markedly protective effect against nephrolithiasis (HR 0.797, 95% CI 0.738–0.861, *p* < 0.001). However, one study evaluating water composition found that drinking from underground water sources, compared to mineral or treated domestic water, was associated with more than double the risk (HR 2.195, 95% CI 1.594–3.021, *p* < 0.001). Regarding alcoholic beverages, moderate-to-high total alcohol intake (11 reports; HR 0.862, 95% CI 0.792–0.937, *p* = 0.001), beer (5 reports; HR 0.817, 95% CI 0.697–0.957, *p* = 0.012), and wine consumption (6 reports; HR 0.839, 95% CI 0.770–0.915, *p* < 0.001) were significantly and inversely correlated with nephrolithiasis risk. In contrast, liquor consumption showed no significant correlation (HR 0.958, 95% CI 0.884–1.038, *p* = 0.293) (Table 1).

Substantial and statistically significant heterogeneity was present across most of these significant exposures. High variance was observed for total water volume (I^2^ = 89.7%, *p* < 0.001), total alcohol (I^2^ = 83.0%, *p* < 0.001), beer (I^2^ = 73.9%, *p* < 0.001), underground water sources (I^2^ = 66.3%, *p* = 0.031), and wine (I^2^ = 56.4%, *p* < 0.001). Conversely, the non-significant findings for liquor demonstrated an absence of heterogeneity (I^2^ = 0%).

### 3.7. Micronutrients, Dietary Acid Load, and Dietary Patterns

Adherence to the Dietary Approaches to Stop Hypertension (DASH) dietary pattern (2 reports) significantly reduced nephrolithiasis risk by 23.9% (HR 0.761, 95% CI 0.715–0.809, *p* < 0.001). Protective effects were also observed for specific micronutrients and dietary components, including dietary calcium (8 reports; HR 0.871, 95% CI 0.842–0.900, *p* < 0.001), potassium (5 reports; HR 0.715, 95% CI 0.669–0.765, *p* < 0.001), phytate (1 report; HR 0.781, 95% CI 0.650–0.938, *p* = 0.008), plant protein (1 report; HR 0.931, 95% CI 0.876–0.989, *p* = 0.020), and niacin (1 report; HR 0.816, 95% CI 0.731–0.911, *p* < 0.001) (Table 1).

Conversely, elevated risk was driven by factors related to dietary acid load and processing, including net endogenous acid production (1 report; HR 1.891, 95% CI 1.537–2.327, *p* < 0.001), potential renal acid load (1 report; HR 1.322, 95% CI 1.077–1.623, *p* = 0.008), and ultra-processed foods (2 reports; HR 1.532, 95% CI 1.105–2.124, *p* = 0.011). Additional risk factors included a high daily insulin index (1 report; HR 1.219, 95% CI 1.079–1.377, *p* = 0.001), sucrose intake (2 reports; HR 1.212, 95% CI 1.101–1.335, *p* < 0.001), high dietary sodium (4 reports; HR 1.129, 95% CI 1.047–1.218, *p* = 0.002), and dietary oxalate (1 report; HR 1.067, 95% CI 1.018–1.118, *p* = 0.007) (Table 1).

Significant statistical heterogeneity was observed among several of the protective dietary components, including the DASH diet (I^2^ = 76.7%, *p* < 0.001), phytate (I^2^ = 74.3%, *p* = 0.009), potassium (I^2^ = 73.7%, *p* < 0.001), and dietary calcium (I^2^ = 54.7%, *p* < 0.001). Among the risk-promoting factors, significant heterogeneity was noted for ultra-processed foods (I^2^ = 86.4%, *p* < 0.001) and high dietary sodium (I^2^ = 68.8%, *p* < 0.001), whereas the acid load metrics (NEAP and PRAL), insulin index, and oxalate exhibited no heterogeneity (I^2^ = 0%).

The direction of association between dietary factors and nephrolithiasis risk classified into risk factors, protective factors, and null associations is shown in Table 2.

### 3.8. Sensitivity Analysis

The results of the sensitivity analysis restricted to high-quality prospective cohorts are summarized in Table 3. A significantly reduced risk of nephrolithiasis persisted for high intakes of fruit (3 reports; HR 0.824, 95% CI 0.788–0.861), dairy products (2 reports; HR 0.918, 95% CI 0.884–0.953), and coffee (4 reports; HR 0.841, 95% CI 0.798–0.887). An increased risk remained significant for sugar-sweetened beverages (1 report; HR 1.302, 95% CI 1.189–1.426) and sodium (2 reports; HR 1.194, 95% CI 1.091–1.308). In contrast, tea (3 reports; HR 0.985, 95% CI 0.915–1.060) and water volume (8 reports; HR 0.961, 95% CI 0.874–1.057) no longer demonstrated significant protective effects, unlike the risk reductions seen in the primary analysis (HR 0.925 and 0.797, respectively). For the outcomes regarding dietary calcium, oxalate, sucrose, and the DASH diet, all included studies were prospective cohorts assessed as having a ‘Low’ or ‘Some concerns’ risk of bias, which significantly increases the robustness of these associations.

### 3.9. Publication Bias

Publication bias was statistically evaluated across all dietary domains using Egger’s regression test. For the majority of analyzed dietary exposures, including major domains such as meat, dairy, coffee, total alcohol, and the DASH dietary pattern, there was no significant evidence of publication bias (Egger’s *p* > 0.05) or small-study effects. However, Egger’s test suggested potential publication bias for a subset of exposures in the binary models, including total vegetables (*p* = 0.007), leafy vegetables (*p* < 0.001), grapefruit juice (*p* = 0.031), punch juice (*p* = 0.006), underground water sources (*p* = 0.019), water hardness (*p* = 0.008), potassium (*p* = 0.007), and phytate (*p* = 0.011). Similar evidence of asymmetry was noted in the continuous models for tea (*p* = 0.038) and fatty acids (*p* = 0.043). These specific findings should be interpreted with caution, as the limited number of available reports for many of these specific variables restricts the statistical power and reliability of Egger’s test to detect true publication bias.

## 4. Discussion

### 4.1. Principal Findings

In this comprehensive meta-analysis, we identified distinct dietary patterns, beverages, and micronutrients that significantly influence the risk of nephrolithiasis. Adherence to a plant-forward approach—specifically the DASH diet, plant proteins, fruits, vegetables, and dietary fiber—alongside dairy products and niacin demonstrated a strong protective effect against stone formation. Furthermore, high total water intake and the consumption of specific beverages, including coffee, tea, orange juice, and moderate alcohol (beer and wine), were associated with reduced risk. Conversely, nephrolithiasis risk was significantly elevated by diets characterized by high renal acid loads, ultra-processed foods, and meat consumption. Additionally, high intakes of sucrose, sodium, oxalate, fatty acids, and sugar-sweetened beverages (including soda, grapefruit juice, and punch), as well as reliance on underground water sources, were identified as notable risk factors. However, the differences in statistical significance observed between the binary and continuous analyses for several dietary factors—including vegetables, dietary fiber, dairy products, caffeine, tea, sugar-sweetened beverages, alcohol, water volume, sodium, and potassium—suggest potential non-linear dose–response relationships that warrant further investigation.

Furthermore, we conducted a sensitivity analysis restricted exclusively to prospective cohorts with a low or moderate risk of bias. The evidence base for dietary calcium, oxalate, sucrose, and the DASH diet is particularly strong, as these were evaluated entirely within high-quality prospective cohorts. Additionally, the fact that significant associations persisted for fruit, dairy, coffee, sodium, and sugar-sweetened beverages underscores the robustness of these dietary factors. Interestingly, the protective effects of tea and water volume observed in the primary analysis lost statistical significance in the restricted prospective cohort model. This attenuation may be attributable to a loss of statistical power due to the reduced number of included studies, as well as the persistently high heterogeneity.

### 4.2. Plant-Based Diets and Dietary Patterns

The protective effects of plant-based foods are largely driven by their favorable impact on urinary chemistry and acid–base balance. Fruits and vegetables are rich sources of potassium, magnesium, and organic anions such as citrate. In the body, these organic anions are metabolized into bicarbonate, providing a net alkali load that raises urine pH and increases urinary citrate excretion [13,14,15]. Citrate is a potent natural inhibitor of nephrolithiasis; it chelates free calcium in the urine, thereby reducing calcium oxalate supersaturation, and prevents the agglomeration of existing crystals [16,17,18]. Furthermore, plant-based diets provide dietary phytate, for which the pooled categorical estimate was inverse but came from one report. Phytate acts as a potent inhibitor of calcium crystallization in the urinary tract [19,20], further reinforcing the benefits of a plant-forward diet.

### 4.3. Dietary Calcium

Our findings demonstrate that both dietary calcium and dairy consumption significantly reduce stone risk. This aligns with prospective evidence associating usual dietary calcium intake with a lower risk of nephrolithiasis [21,22,23,24]. Dietary calcium consumed through dairy products or calcium-rich foods effectively binds to dietary oxalate within the intestinal tract, forming an insoluble calcium-oxalate complex that is excreted in the feces [20,22]. This intestinal binding prevents the absorption of free oxalate into the bloodstream, consequently lowering urinary oxalate excretion [25,26]. However, when calcium supplements are taken in isolation between meals, the calcium is readily absorbed without chelating oxalate, thereby increasing the urinary calcium load and potentially raising the risk of stone formation. This mechanism is supported by a short crossover study demonstrating that while calcium supplementation increased urinary calcium regardless of timing, only supplements taken simultaneously with meals successfully reduced urinary oxalate [27].

### 4.4. Fluids and Beverages

Adequate fluid intake remains an established part of stone-prevention care, although the restricted prospective categorical estimate for reported water volume included the null and showed very high heterogeneity. Increased total water volume mechanically dilutes calculogenic urinary solutes—such as calcium, oxalate, and uric acid—thereby lowering their supersaturation [28]. Furthermore, higher urine flow rates decrease the transit time of solutes through the renal tubules, minimizing the opportunity for crystal nucleation, growth, and aggregation [29].

Beyond simple hydration, specific beverages exert unique physiological effects. Although caffeine exerts a hypercalciuric effect [30], its diuretic actions, mediated in part through competitive antagonism of adenosine A1 receptors in renal proximal tubular cells [31], together with the suppression of Na^+^/K^+^-ATPase and Na^+^/H^+^ exchanger activities and the enhancement of renal atrial natriuretic peptide (ANP) and nitric oxide (NO) activity [32], may confer protective effects against nephrolithiasis. In vitro evidence suggests that caffeine’s protective mechanism against nephrolithiasis is a reduction of calcium oxalate crystal adhesion on the apical surface of renal tubular epithelial cells [33]. While tea contains naturally occurring oxalate, our findings suggest that its diuretic effect and its rich concentration of antioxidant polyphenols [34,35] may affect urinary chemistry, but the prospective categorical tea estimate included the null. Similarly, moderate consumption of alcohol, particularly beer and wine, was inversely associated with risk. Alcohol suppresses the release of antidiuretic hormone (vasopressin) from the posterior pituitary, leading to significant diuresis [36]. Additionally, wine provides fluid alongside alkali loads and antioxidants [37]. Orange juice had an inverse estimate in one report, likely due to its high potassium and citrate content, which effectively alkalinizes the urine and chelates urinary calcium [38,39].

In contrast, sugar-sweetened beverages, soda, and certain fruit juices (such as grapefruit and punch juice) significantly elevated nephrolithiasis risk. These beverages are typically high in fructose and sucrose. The metabolism of dietary fructose increases urinary calcium excretion, promotes uric acid synthesis, and may increase urinary oxalate [40]. Furthermore, high consumption of refined sugars contributes to hyperinsulinemia and insulin resistance [41], which can impair renal ammonia production and lower urine pH, creating an ideal environment for uric acid precipitation [42].

### 4.5. Animal Protein, Sodium, and Acid Load

Higher meat intake and dietary acid-load indices showed positive associations in our study. Meat and other animal-derived foods are particularly rich in sulfur-containing amino acids, such as methionine and cysteine, which contribute to a higher dietary acid load, as reflected by increased net endogenous acid production (NEAP) and potential renal acid load (PRAL) [43,44]. PRAL is inversely correlated with urinary citrate, with higher PRAL associated with lower urinary citrate excretion [45]. Moreover, a high-protein diet has been associated with increased urinary oxalate [3], net acid excretion, and urinary calcium, accompanied by reductions in urinary pH and citrate [4]. Additionally, the high purine content in animal protein increases both the production and urinary excretion of uric acid; when combined with a lowered urine pH, this strongly favors the precipitation of uric acid stones. Collectively, these alterations in urinary composition may increase the risk of uric acid and calcium oxalate crystals.

Sodium may increase urinary calcium excretion; the positive categorical association persisted in two prospective reports [6,46]. The combined clinical effects of meat, sodium, and ultra-processed foods cannot be inferred from these separate observational estimates.

### 4.6. Strengths

A strength of this systematic review and meta-analysis is its comprehensive scope. This broad approach allows for a holistic understanding of how various dietary patterns, beverages, and specific nutrients interact to influence stone risk. Furthermore, by strictly extracting multivariable-adjusted effect sizes, we minimized the influence of confounding variables, thereby strengthening the reliability of the observed associations.

### 4.7. Limitations

Several limitations constrain the interpretation of our findings. First, pooling incident, recurrent, and prevalent stone outcomes alongside cross-sectional and retrospective dietary assessments introduces a risk of reverse causation, as patients may alter their dietary habits following a stone diagnosis. Although our sensitivity analysis restricted to high-quality prospective cohorts mitigates this temporal bias and reinforces our core findings, the observational nature of the underlying data ultimately precludes establishing definitive causality. Second, we observed massive statistical heterogeneity across several key pooled estimates—most notably for total water volume, total alcohol, and the DASH diet. Because of the limited number of primary studies available for these specific exposures, we were unable to perform meta-regression or extensive subgroup analyses to investigate the sources of this variance; therefore, while the protective direction of these diets is clear, the exact magnitude of clinical benefit may vary. Third, repeated reports derived from the same cohorts, as well as multiple contrasts sharing a common reference group, may produce dependent estimates; treating these as independent variables could potentially overstate statistical precision. Finally, as is inherent to all nutritional meta-analyses, our conclusions rely heavily on the methodological integrity of the primary studies. The predominant use of self-reported outcomes, variable covariate adjustments, and dietary assessments via food frequency questionnaires (FFQs) or dietary recalls renders the data susceptible to measurement error, selective reporting, and recall bias.

### 4.8. Clinical Implications

Diet plays a profound and highly modifiable role in the pathogenesis of nephrolithiasis. The findings of this meta-analysis offer evidence-based guidelines for dietary counseling in the prevention and management of nephrolithiasis.

Clinicians should transition away from restrictive, single-nutrient paradigms and instead emphasize holistic dietary patterns. Specifically, counseling should focus on the following core principles. First, adequate hydration through increased total fluid intake remains the cornerstone of stone prevention. While coffee and tea can be safely consumed, sugar-sweetened beverages, sodas, and high-fructose juices must be strictly limited. Second, patients should increase their intake of foods with a high alkali load by adopting a DASH-style or plant-forward diet rich in fruits, vegetables, and plant proteins. This approach naturally provides the potassium, citrate, and phytate necessary to increase urine pH and inhibit crystal formation. Third, normal calcium intake should be maintained to ensure adequate intestinal binding of oxalate and prevent enteric hyperoxaluria. Dairy products can be consumed without increasing the risk of nephrolithiasis. Finally, dietary interventions must prioritize the reduction in net endogenous acid production (NEAP) by limiting the intake of animal protein, ultra-processed foods, and dietary sodium. Crucially, the protective effects of fruit, dairy, and coffee, alongside the risks associated with sodium and sugar-sweetened beverages, were proven highly robust in our strict sensitivity analysis of high-quality prospective cohorts, making them exceptionally reliable targets for patient counseling. However, clinicians must interpret and apply these recommendations with caution. Given the massive statistical heterogeneity observed across several key exposures—most notably total water volume and the DASH diet—the exact magnitude of clinical benefit will likely vary considerably among individuals. While the overall protective direction of these dietary patterns is robust, counseling must be individualized, and these interventions should not be framed as yielding uniform risk reductions for all patients.

## 5. Conclusions and Future Prospects

This systematic review and meta-analysis comprehensively evaluates a complex array of diverse dietary factors influencing nephrolithiasis formation, distinguishing itself by assessing holistic dietary patterns rather than focusing solely on individual nutritional components. This study demonstrates that a plant-forward, alkali-promoting dietary pattern—such as the DASH diet—coupled with high fluid volume, adequate dairy consumption, and caffeinated beverages, provides robust protection against stone formation. Conversely, Western dietary habits characterized by high meat consumption, ultra-processed foods, refined sucrose, and sugar-sweetened beverages significantly elevate stone risk by driving unfavorable urinary chemistry and high dietary acid loads. Ultimately, nutritional strategies for nephrolithiasis prevention should prioritize holistic, balanced dietary patterns that maximize natural crystallization inhibitors while minimizing systemic acid and sodium burdens. Future research should prioritize evaluating comprehensive dietary interventions for stone prevention and standardizing dietary assessment tools across diverse clinical populations.

## Figures and Tables

**Table 1:** Summary risk estimates of the association between dietary factors and risk of kidney stones.

Food/Exposure	Model	No. of Studies	No. of Study Arms	HR (95% CI)	*p*-Value	I^2^ (%)	Q Test *p*-Value	Egger’s Test *p*-Value
Plant-based and dietary fiber								
Fruit	Adjusted Binary (high vs. low)	6	36	0.849 (0.818–0.881)	<0.001	35.817	0.019	0.107
Vegetable	Adjusted Binary (high vs. low)	3	13	0.910 (0.839–0.987)	0.023	49.387	0.022	0.0071
Vegetable	Adjusted continuous (grams/day)	1	3	0.959 (0.852–1.081)	0.494	0	0.873	0.419
Dietary Fiber	Adjusted Binary (high vs. low)	2	12	0.825 (0.766–0.889)	<0.001	70.153	<0.001	0.067
Dietary Fiber	Adjusted continuous (milligrams/day)	3	6	0.948 (0.848–1.06)	0.35	88.054	<0.001	0.407
Leafy Vegetable	Adjusted Binary (frequent vs. non-frequent)	2	9	1.094 (0.957–1.250)	0.19	73.605	<0.001	<0.001
Grains	Adjusted Binary (high vs. low)	2	10	1.123 (0.954–1.322)	0.163	0	0.462	0.199
Beans/Legumes	Adjusted Binary (frequent vs. non-frequent)	2	7	1.297 (0.869–1.938)	0.203	73.895	0.001	0.218
Tubers	Adjusted Binary (frequent vs. non-frequent)	1	6	1.190 (0.981–1.443)	0.078	0	0.974	0.3
Root-Stock Food	Adjusted Binary (frequent vs. non-frequent)	2	7	1.078 (0.924–1.258)	0.34	0	0.34	0.727
Nuts	Adjusted Binary (frequent vs. non-frequent)	1	4	1.038 (0.886–1.216)	0.646	0	0.714	0.378
Betel nut	Adjusted Binary (high vs. low)	1	4	1.132 (0.994–1.289)	0.061	54.787	0.084	0.498
Animal-based foods and protein sources								
Meat	Adjusted Binary (high vs. low)	2	8	1.153 (1.100–1.209)	<0.001	0	0.597	0.341
Dairy Products	Adjusted Binary (frequent vs. non-frequent)	4	27	0.926 (0.886–0.968)	0.001	23.66	0.134	0.48
Dairy Products	Adjusted continuous (servings/day)	3	5	0.949 (0.885–1.017)	0.141	0	0.864	0.055
Fish	Adjusted Binary (high vs. low)	2	10	1.020 (0.991–1.050)	0.171	0	0.815	0.719
Fish	Adjusted continuous	2	5	1.012 (0.982–1.043)	0.423	29.554	0.225	0.316
Eggs	Adjusted Binary (frequent vs. non-frequent)	2	7	0.870 (0.647–1.170)	0.356	33.167	0.175	0.212
Meat & Fish	Adjusted Binary (frequent vs. non-frequent)	2	7	0.821 (0.663–1.017)	0.071	0	0.57	0.567
Meat & Fish	Adjusted continuous	2	2	1.006 (0.99–1.023)	0.468	43.85	0.182	NA
Animal Protein	Adjusted Binary (high vs. low)	6	64	1.006 (0.983–1.029)	0.62	38.609	0.001	0.239
Animal Protein	Adjusted continuous (grams/day)	3	5	0.997 (0.992–1.002)	0.18	0	0.771	0.517
Non-alcoholic beverages, juices, and sugar-sweetened beverages								
Coffee	Adjusted Binary (high vs. low)	6	30	0.845 (0.803–0.890)	<0.001	54.88	<0.001	0.812
Coffee	Adjusted Continuous (serving/day)	3	5	0.909 (0.887–0.932)	<0.001	0	0.954	0.109
Caffeine	Adjusted Binary (high vs. low)	2	27	0.909 (0.850–0.971)	0.005	68.4	<0.001	0.371
Caffeine	Adjusted Continuous (milligram/day)	2	4	0.974 (0.825–1.149)	0.753	67.194	0.027	0.477
Tea	Adjusted Binary (high vs. low)	12	38	0.925 (0.874–0.980)	0.008	70.469	<0.001	0.818
Tea	Adjusted Continuous (serving/day)	4	4	0.868 (0.772–0.976)	0.018	84.856	<0.001	0.038
Orange Juice	Adjusted Binary (high vs. low)	1	4	0.903 (0.851–0.958)	0.001	0	0.877	0.335
Orange Juice	Adjusted Continuous (serving/day)	2	2	0.967 (0.829–1.129)	0.675	0	0.696	NA
Sugar-Sweetened Beverages	Adjusted Binary (high vs. low)	2	12	1.340 (1.251–1.436)	<0.001	56.642	0.008	0.708
Sugar-Sweetened Beverages	Adjusted Continuous	1	2	1.038 (0.99–1.088)	0.12	92.036	<0.001	NA
Soda	Adjusted Binary (high vs. low)	2	20	1.067 (1.003–1.135)	0.039	50.908	0.005	0.248
Soda	Adjusted Continuous (serving/day)	2	8	1.008 (0.968–1.048)	0.709	11.729	0.339	0.544
Grapefruit Juice	Adjusted Binary (high vs. low)	3	12	1.116 (1.033–1.207)	0.006	11.515	0.332	0.031
Grapefruit Juice	Adjusted Continuous (serving/day)	2	2	1.407 (1.144–1.73)	0.001	0	0.814	NA
Punch Juice	Adjusted Binary (high vs. low)	1	4	1.141 (1.067–1.220)	<0.001	0	0.777	0.006
Punch Juice	Adjusted Continuous (serving/day)	2	2	1.011 (0.832–1.228)	0.913	70.178	0.067	NA
Apple Juice	Adjusted Binary (high vs. low)	2	8	1.009 (0.917–1.110)	0.855	17.21	0.294	0.361
Apple Juice	Adjusted Continuous (serving/day)	2	2	1.009 (0.513–1.985)	0.979	75.864	0.042	NA
Tomato Juice	Adjusted Binary (high vs. low)	1	4	1.01 (0.861–1.185)	0.902	0	0.951	0.376
Tomato Juice	Adjusted Continuous (serving/day)	1	2	1.35 (0.851–2.141)	0.202	0	0.838	NA
Alcohol								
Total Alcohol	Adjusted Binary (high vs. low)	11	25	0.862 (0.792–0.937)	0.001	83.032	<0.001	0.969
Total Alcohol	Adjusted Continuous	5	5	0.829 (0.515–1.334)	0.44	95.574	<0.001	0.9
Beer	Adjusted Binary (high vs. low)	5	18	0.817 (0.697–0.957)	0.012	73.902	<0.001	0.384
Beer	Adjusted Continuous (serving/day)	2	2	0.81 (0.732–0.895)	<0.001	0	0.376	NA
Wine	Adjusted Binary (high vs. low)	6	26	0.839 (0.770–0.915)	<0.001	56.359	<0.001	0.167
Wine	Adjusted Continuous (serving/day)	2	2	0.518 (0.353–0.760)	0.001	34.773	0.216	NA
Liquor	Adjusted Binary (high vs. low)	3	9	0.958 (0.884–1.038)	0.293	0		0.166
Liquor	Adjusted Continuous (serving/day)	2	2	0.807 (0.492–1.325)	0.396	0	0.536	NA
Water and hydration parameters								
Water Volume Intake	Adjusted Binary (high vs. low)	11	41	0.797 (0.738–0.861)	<0.001	89.74	<0.001	0.456
Water Volume Intake	Adjusted Continuous (milliliter/day)	6	9	0.978 (0.929–1.03)	0.396	56.27	0.019	0.819
Underground Water Source	Adjusted Binary (high vs. low)	1	4	2.195 (1.594–3.021)	<0.001	66.3	0.031	0.019
Water Hardness	Adjusted Binary (high vs. low)	2	10	0.929 (0.835–1.032)	0.171	56.92	0.013	0.008
Water Ca Concentration	Adjusted Binary (high vs. low)	1	6	1.039 (0.996–1.084)	0.078	0	0.779	0.074
Water Mg Concentration	Adjusted Binary (high vs. low)	1	3	1.040 (0.979–1.105)	0.208	0	0.76	0.932
Dietary minerals and micronutrients								
Dietary Calcium	Adjusted Binary (high vs. low)	7	60	0.9 (0.861–0.941)	<0.001	74.678	<0.001	0.005
Dietary Calcium	Adjusted Continuous (milligram/day)	1	4	0.735 (0.519–1.041)	0.083	82.878	0.001	NA
Potassium	Adjusted Binary (high vs. low)	5	28	0.715 (0.669–0.765)	<0.001	73.67	<0.001	0.007
Potassium	Adjusted Continuous (milligram/day)	2	4	0.781 (0.6–1.018)	0.068	89.463	<0.001	0.068
Phytate	Adjusted Binary (high vs. low)	1	4	0.781 (0.650–0.938)	0.008	74.33	0.009	0.011
Phytate	Adjusted Continuous (milligram/day)	1	3	0.888 (0.73–1.08)	0.233	59.525	0.085	0.119
Niacin	Adjusted Binary (high vs. low)	1	3	0.816 (0.731–0.911)	<0.001	0	0.877	0.455
Sodium	Adjusted Binary (high vs. low)	4	15	1.129 (1.047–1.218)	0.002	68.802	<0.001	0.665
Sodium	Adjusted Continuous (milligram/day)	2	3	0.963 (0.858–1.08)	0.517	0	0.649	0.317
Dietary Oxalate	Adjusted Binary (high vs. low)	1	12	1.067 (1.018–1.118)	0.007	0	0.631	0.271
Dietary Oxalate	Adjusted Continuous (milligram/day)	1	3	1.055 (0.947–1.176)	0.331	0	0.657	0.273
Zinc	Adjusted Binary (high vs. low)	2	10	0.848 (0.646–1.113)	0.235	60.919	0.006	0.298
Zinc	Adjusted Continuous (milligram/day)	2	2	1.135 (0.772–1.668)	0.519	89.177	0.002	NA
Macronutrients and specific nutrients								
Plant Protein	Adjusted Binary (high vs. low)	1	4	0.931 (0.876–0.989)	0.02	0	0.782	0.259
Plant Protein	Adjusted Continuous (gram/day)	1	3	0.959 (0.852–1.081)	0.494	0	0.873	0.419
Sucrose	Adjusted Binary (high vs. low)	2	8	1.212 (1.101–1.335)	<0.001	35.655	0.144	0.171
Carbohydrate	Adjusted Binary (high vs. low)	2	2	1.386 (0.985–1.95)	0.061	0	0.333	NA
Oil	Adjusted Binary (frequent vs. non-frequent)	1	4	1 (0.752–1.33)	0.999	26.213	0.254	0.358
Fatty Acids	Adjusted Binary (high vs. low)	1	36	1.021 (0.981–1.062)	0.31	35.274	0.021	0.231
Fatty Acids	Adjusted Continuous (milligram/day)	1	3	1.177 (1.104–1.255)	<0.001	24.866	0.264	0.043
Dietary pattern, acid load and ultra-processed food								
DASH Diet	Adjusted Binary (high vs. low)	2	24	0.761 (0.715–0.809)	<0.001	76.671	<0.001	0.109
Net Endogenous Acid (NEAP)	Adjusted Binary (high vs. low)	1	6	1.891 (1.537–2.327)	<0.001	0	0.997	0.087
Renal Acid Load (PRAL)	Adjusted Binary (high vs. low)	1	6	1.322 (1.077–1.623)	0.008	0	0.932	0.678
Ultra-processed Food	Adjusted Binary (high vs. low)	2	4	1.532 (1.105–2.124)	0.011	86.418	<0.001	0.119
Daily Insulin Index	Adjusted Binary (high vs. low)	1	6	1.219 (1.079–1.377)	0.001	0	0.909	0.568
Soup Consumption	Adjusted Binary (frequent vs. non-frequent)	2	7	0.797 (0.653–0.972)	0.025	0	0.51	0.652
Guangzhou Dessert	Adjusted Binary (frequent vs. non-frequent)	1	6	0.838 (0.705–0.997)	0.046	0	0.884	0.406

Abbreviation: NA, not applicable.

**Table 2:** Categorical summary of dietary factors by direction of association with nephrolithiasis risk.

Food Category	Risk Factors	Protective Factors	Null Association
Plant-based and dietary fiber	-	Fruit (6) Vegetable (3) Dietary fiber (2)	Leafy vegetable (2) Grains (2) Beans/legumes (2) Tubers (1) Root-stock food (2) Nuts (1) Betel nut (1)
Animal-based foods and protein sources	Meat (2)	Dairy products (4)	Fish (2) Eggs (2) Meat and fish (2) Animal protein (6)
Non-alcoholic beverages and juices	Sugar-sweetened beverages (2) Soda (2) Grapefruit juice (3) Punch juice (1)	Coffee (6) Caffeine (2) Tea (12) Orange juice (1)	Apple juice (2) Tomato juice (1)
Alcohol	-	Total alcohol (11) Beer (5) Wine (6)	Liquor (3)
Water and hydration	Underground water source (1)	Water volume intake (11)	Water hardness (2) Water calcium concentration (1) Water magnesium concentration (1)
Dietary minerals and micronutrients	Sodium (4) Dietary oxalate (1)	Dietary calcium (8) Potassium (5) Phytate (1) Niacin (1)	Zinc (2)
Macronutrients	Sucrose (2) Fatty acids (1)	Plant protein (1)	Carbohydrate (2) Oil (1)
Dietary pattern, acid load and ultra-processed food	Net endogenous acid production (NEAP) (1) Potential renal acid load (PRAL) (1) Ultra-processed food (2) Daily insulin index (1)	DASH diet (2) Soup consumption (2) Guangzhou dessert (1)	-

Note: Risk and protective factors are pooled estimates reaching statistical significance (*p* < 0.05); null-association factors were not significant (*p* > 0.05). Numbers in parentheses indicate the number of included studies contributing to each pooled estimate.

**Table 3:** Sensitivity analysis restricted to high-quality prospective cohort studies.

Food/Exposure	Model	No. of Studies	No. of Study Arms	HR (95% CI)	*p*-Value	I^2^ (%)	Q Test *p*-Value	Egger’s Test *p*-Value
Plant-based and dietary fiber								
Fruit	Adjusted Binary (high vs. low)	3	16	0.824 (0.788–0.861)	<0.001	35.237	0.081	0.642
Vegetable	Adjusted Binary (high vs. low)	1	8	0.878 (0.811–0.95)	0.001	47.705	0.063	NA
Dietary Fiber	Adjusted continuous (milligrams/day)	2	3	0.836 (0.779–0.898)	<0.001	5.089	0.349	NA
Leafy Vegetable	Adjusted Binary (frequent vs. non-frequent)	1	3	0.922 (0.877–0.97)	0.002	0	0.818	NA
Animal-based foods and protein sources								
Meat	Adjusted Binary (high vs. low)	1	4	1.153 (1.089–1.22)	<0.001	23.525	0.27	NA
Dairy Products	Adjusted Binary (frequent vs. non-frequent)	2	13	0.918 (0.884–0.953)	<0.001	10.53	0.34	0.406
Fish	Adjusted Binary (frequent vs. non-frequent)	1	9	1.023 (0.995–1.051)	0.11	0	0.744	NA
Animal Protein	Adjusted Binary (high vs. low)	5	24	0.99 (0.951–1.031)	0.645	44.212	0.011	0.412
Non-alcoholic beverages, juices, and sugar-sweetened beverages								
Coffee	Adjusted Binary (high vs. low)	4	25	0.841 (0.798–0.887)	<0.001	57.962	<0.001	0.883
Caffeine	Adjusted Binary (high vs. low)	1	24	0.890 (0.834–0.95)	<0.001	67.54	<0.001	0.564
Tea	Adjusted Binary (high vs. low)	3	9	0.985 (0.915–1.06)	0.686	73.087	<0.001	NA
Tea	Adjusted Continuous (serving/day)	3	3	0.923 (0.876–0.972)	0.002	45.347	0.16	NA
Sugar-Sweetened Beverages	Adjusted Binary (high vs. low)	1	6	1.302 (1.189–1.426)	<0.001	68.512	0.007	NA
Soda	Adjusted Binary (high vs. low)	1	16	1.099 (1.045–1.155)	<0.001	26.472	0.157	0.433
Alcohol								
Total Alcohol	Adjusted Binary (high vs. low)	1	4	0.633 (0.518–0.773)	<0.001	90.761	<0.001	NA
Beer	Adjusted Binary (high vs. low)	2	8	0.809 (0.673–0.973)	0.025	73.287	<0.001	NA
Wine	Adjusted Binary (high vs. low)	3	17	0.851 (0.789–0.917)	<0.001	40.207	0.044	<0.001
Liquor	Adjusted Binary (high vs. low)	1	4	0.923 (0.843–1.011)	0.085	0	0.579	NA
Water and hydration parameters								
Water Volume Intake	Adjusted Binary (high vs. low)	8	33	0.961 (0.874–1.057)	0.417	92.972	<0.001	0.008
Water Volume Intake	Adjusted Continuous (milliliter/day)	4	5	0.983 (0.961–1.006)	0.147	0	0.855	NA
Water Hardness	Adjusted Binary (high vs. low)	1	4	0.979 (0.933–1.027)	0.392	0	0.648	NA
Dietary minerals and micronutrients								
Potassium	Adjusted Binary (high vs. low)	3	20	0.697 (0.639–0.76)	<0.001	78.272	<0.001	0.016
Sodium	Adjusted Binary (high vs. low)	2	8	1.194 (1.091–1.308)	<0.001	42.114	0.098	NA

Abbreviation: NA, not applicable.

## Data Availability

The data presented in this study are available on request from the corresponding author.

## Supplementary Materials

The following supporting information can be downloaded at: https://www.mdpi.com/article/10.3390/nu18213653/s1, Table S1: Search strategies including the queries for each database. Table S2: Characteristics of studies included in the systematic review and meta-analysis [21,23,24,47,48,49,50,51,52,53,54,55,56,57,58,59,60,61,62,63,64,65,66,67,68,69,70,71,72,73,74,75,76,77,78,79,80,81,82,83,84,85,86,87,88,89,90,91,92,93,94,95,96,97,98,99,100,101,102,103,104]. Figure S1: Study Workflow.
