# Supplementary material for: Association of Dietary Factors with Nephrolithiasis: A Systematic Review and Meta-Analysis
Source: Nutrients. 2026 Nov 3;18(21):3653. doi: 10.3390/nu18213653 (PMC13765395; doi:10.3390/nu18213653)

**Supplementary Table S1** Search strategies including the queries for each database

| **Database** | **key terms and the queries** |
|---|---|
| PubMed | #1 ((((((((fluid[Text Word]) OR (diet[Text Word])) OR (dietary[Text Word])) OR (fruit[Text Word])) OR (vegetables[Text Word])) OR (vegetable[Text Word])) OR (dietary fiber[Text Word])) OR (diet[MeSH Terms])) OR (dietary[MeSH Terms])<br><br>#2 ((((nephrolithiasis[Text Word]) OR (kidney stones[Text Word])) OR (urinary calculi[Text Word])) OR (renal calculi[Text Word])) OR (nephrolithiasis[MeSH Terms])<br><br>#3 (risk factor*[Text Word]) OR (risk factors[MeSH Terms])<br><br>#4 #1 AND #2 AND #3 |
| Scopus | #1 ( TITLE-ABS-KEY ( "fluid" ) OR TITLE-ABS-KEY ( "diet" ) OR TITLE-ABS-KEY ( "dietary" ) OR TITLE-ABS-KEY ( "fruit" ) OR TITLE-ABS-KEY ( "vegetables" ) OR TITLE-ABS-KEY ( "vegetable" ) OR TITLE-ABS-KEY ( "dietary fiber" ) )<br>#2 ( TITLE-ABS-KEY ( "nephrolithiasis" ) OR TITLE-ABS-KEY ( "kidney stones" ) OR TITLE-ABS-KEY ( "urinary calculi" ) OR TITLE-ABS-KEY ( "renal calculi" ) )<br>#3 ( TITLE-ABS-KEY ( "risk factor" ) OR TITLE-ABS-KEY ( "risk factors" ) )<br>#4 ( ( TITLE-ABS-KEY ( "risk factor" ) OR TITLE-ABS-KEY ( "risk factors" ) ) ) AND ( ( TITLE-ABS-KEY ( "nephrolithiasis" ) OR TITLE-ABS-KEY ( "kidney stones" ) OR TITLE-ABS-KEY ( "urinary calculi" ) OR TITLE-ABS-KEY ( "renal calculi" ) ) ) AND ( ( TITLE-ABS-KEY ( "fluid" ) OR TITLE-ABS-KEY ( "diet" ) OR TITLE-ABS-KEY ( "dietary" ) OR TITLE-ABS-KEY ( "fruit" ) OR TITLE-ABS-KEY ( "vegetables" ) OR TITLE-ABS-KEY ( "vegetable" ) OR TITLE-ABS-KEY ( "dietary fiber" ) ) ) |

**Supplementary Table S2** Characteristics of studies included in the systematic review and meta-analysis

| Author | Study design | Sample size | Follow-up period | Mean age (yr) | Male, n (%) | Exposure type | Exposure assessment | Outcome assessment | Risk of bias |
|---|---|---|---|---|---|---|---|---|---|
| Xiangyu Zhang, 2025 [47] | cross-sectional analysis | 21,547 | No | 50.96 ± 17.31 | 10,391 (48.2) | Meat, oily fish, non-oily fish | Dietary survey data | Questionnaire "Have you ever had kidney stones?" | High risk |
| Kamran Hassan Bhatti, 2025 [48] | Retrospective cross-sectional study | 2,800 | No | 43.4 ± 12.5 | 2,363 (84.4) | Alcohol | Medical records | Having kidney stone | High risk |
| Peng Chen, 2025 [49] | cross-sectional analysis | 15,779 | No | 40.1 ± 11.87 | 7,921 (50.2) | Sugar sweetened beverage (sodas, carbonated water, energy drinks, fruit drinks, soft drinks, smoothies, sports drinks, sugar-sweetened coffee and tea) | 24-hour dietary recall interviews | Questionnaire "Have you ever had kidney stones?" | High risk |
| Song Bai, 2025 [50] | prospective cohort | 166,427 | Mean 9.07 years | 55.6 (7.9) (no stone), 56.2 (7.6) (stone) | 74,652 (44.9) | Ultra-processed food | Web-based 24-hour dietary assessment tool | ICD-10, the Office of Population Censuses and Surveys Classification of Interventions and Procedures, version 4 (OPCS-4), and a single self-reported operation code for kidney stone surgery | Some concerns |

| | | | | | | | | | |
|---|---|---|---|---|---|---|---|---|---|
| Jian Wu, 2025 [51] | cross-sectional study | 28,508 | No | 47.93 ± 0.24 | 13,402 (46.5) | Niacin | First 24-hour dietary recalls interview, telephone interview between 3 and 10 days later | Questionnaire “Have you ever had kidney stones?” | Very high risk |
| Jiahao Zhang, 2025 [52] | prospective cohort | 288,041 | Median 15.1 year | 55.51 ± 2.82 | 131,407 (45.6) | Domestic water hardness, Magnesium concentration, Calcium carbonate and Calcium concentration | Domestic water hardness was collected in 2 rounds, including surveys in 2005 and 2013 | Hospital inpatient records | Low risk |
| Ahmad Delbari, 2025 [53] | Cross-sectional study | 4,884 | NA | NA | 2,349 (48.1) | Alcohol | Questionnaire "Have you ever consumed alcohol?” | Presence of solid deposits comprised of minerals and salts in the kidneys, causing considerable pain and discomfort during passage through the urinary tract. Participants were asked to self-report whether they had received a medical diagnosis of kidney stones. | High risk |
| Ning Tan, 2025 [54] | Cross-sectional study | 30,716 | No | 49.69 ± 17.69 | 14,940 (48.6) | Saturated fatty acid, monounsaturated fatty acid, polyunsaturated fatty acid | 24-hour dietary recall interview | Questionnaire "Have you or the sample person ever had a kidney stones?" | High risk |
| Kuan-Hsien Wu, 2024 [55] | Cross-sectional study | 11,484 | No | 60.82 ± 6.01 | 0 (0) | Tea | Questionnaire | Questionnaire | Very high risk |

| | | | | | | | | | |
|---|---|---|---|---|---|---|---|---|---|
| Sandipan Shringi, 2024 [56] | Cross-sectional study | 29,684 | No | 53.7 ± 0.38 (Kidney stone former), 46.8 ± 0.26 (non-kidney stone former) | 14,436 (48.6) | Beer, wine, liquor | 24-hour dietary recall interviews | Interview question 'Have you ever had a kidney stone?' | High risk |
| Xiaoqin Gan, 2024 [57] | Prospective cohort | 205,896 | 11.6 years (11.0–12.4 years) (interquartile range) | 56.1 (SD 7.9) | 91,677 (44.5) | Total fruits, citrus, pomes, tropical fruit | Web-based 24-hour dietary recall questionnaire (Oxford WebQ) | Data on hospital admissions, operations, and procedures, which was identified using the International Classification of Diseases (ICD) | Some concerns |
| Jie Tang, 2024 [58] | Cross-sectional study | 19,405 | No | 54 (±0.4) (stone former), 47 (±0.3) (non-stone former) | 9,215 (47.5) | Sodium, potassium | 24-hour recall queried all foods/beverages consumed from midnight-to-midnight on the day before the interview | Interview data file of "Have you ever had a kidney stone?" | High risk |
| Bahman Cheraghian, 2024 [59] | Population-based cross-sectional study | 10,009 | No | 48.76 ± 9.21 | 4,026 (40.3) | Protein, fat, fiber, carbohydrate, calcium, iron, magnesium, potassium, copper, zinc, fluoride, manganese, vitamin B12, vitamin E, vitamin K, vitamin A, vitamin B6, vitamin D, folate | Semi-quantitative food frequency questionnaire (FFQ) | Participants self-reported a history of kidney stones if the disease had been previously diagnosed by a doctor. All medical documents, including ultrasounds, photographs, | High risk |

| | | | | | | | | | |
|---|---|---|---|---|---|---|---|---|---|
| | | | | | | | | laboratory reports, and surgical records | |
| Zhilong Li, 2024 [60] | Two-sample Mendelian Randomization (MR) study | 341,725 | NA | NA | NA | Alcohol, tea | GWAS database | FinnGen consortium | Some concerns |
| Widi Atmoko, 2024 [61] | Retrospective cohort | 325 | Mean 30.7 (3.2) months | 52 (18-80) | 176 (54.1) | Fluid intake, tea consumption, vegetable consumption, lean/fatty meat consumption | Medical records, interview, and online questionnaires | Medical records | Some concerns |
| Constance Legay, 2023 [62] | Case-control | 458 | NA | NA | 275 (60) | Potato, fruits, nuts and seeds, milk, yogurt, cheese, meat, fish and seafood, eggs, sugar/chocolate, cakes and biscuits, juices, soft drinks, coffee, tea, infusion, wine, beer, soup, savory snacks | Two consecutive 24-hour dietary recalls | Cases were clinically confirmed patients from dedicated kidney stone centers | High risk |
| Yi-Hsuan Chen, 2023 [63] | Longitudinal cohort study | 25,256 | 4 | 51 ± 10 | 8,587 (34) | Alcohol | Questionnaires | Questionnaire "Have you ever had kidney stone disease?" | High risk |
| Sara Shojaei-Zarghani, 2023 [64] | Cross-sectional study | 4,901 | NA | 48.14 | 2,386 (48.7) | Alcohol | General and medical questionnaires | Questionnaire: "Have you ever had nephrolithiasis?", "Are you being treated for nephrolithiasis?" | High risk |
| Maria D'Amico, 2023 [65] | Retrospective cohort | 43,178 | NA | 51.9 ± 10.3 (ever reported | 0 (0) | Alcohol | Questionnaires | Questionnaires | High risk |

| | | | | | | | | | |
|---|---|---|---|---|---|---|---|---|---|
| | | | | nephrolithiasis) 49.2 ± 10.7 (never reported nephrolithiasis) | | | | | |
| Hicham I. Cheikh Hassan, 2023 [66] | Retrospective population-based cohort study | 36,138 | Mean 4.8 ± 3.1 year | 70 (16.6) (no AKI), 68.4 (16.7) (AKI) | 17,522 (48.5) | Alcohol | Medical records | Inpatient ICD-10-AM coding and from Systemized Nomenclature Of Medicine (SNOMED) emergency coding | Some concerns |
| So Young Kim, 2022 [67] | Case-control | 141,975 | NA | NA | 91,300 (64.3) | Alcohol | Self-reported survey during health checkup | Nephrolithiasis (ICD-10 codes: N20) was defined if the participants treated it ≥2 times | High risk |
| Yingyu Liu, 2022 [68] | Case-control | 834 | NA | 53.87 (53.01–54.74) (mean, 95% CI) | 557 (66.8) | Tea, green tea, black tea | 100-item validated semiquantitative food frequency questionnaire (FFQ) with specified serving sizes | Hospitalized nephrolithiasis requiring surgical intervention (identified via ICD-10 codes N20) | Some concerns |
| Api Chewcharat, 2022 [69] | Prospective case-control study | 795 | Median 4.1 (1.3-5.6) years | 47.3 ± 14.4 | 407 (51.2) | Calcium, fluid, sodium, potassium, animal protein, vegetable protein, caffeine, fiber, phytate, oxalate | Electronic VioScreen food frequency questionnaire (FFQ; Viocare, Inc) | Kidney stone diagnostic codes and clinical notes for the term kidney stone | Some concerns |

| | | | | | | | | | |
|---|---|---|---|---|---|---|---|---|---|
| Chun-Kai Chang, 2022 [70] | Cross-sectional cohort | 43,636 | No | 50 ± 11 | 43,636 (100) | Betel nut | Questionnaire “Have you ever had experience of betel nut chewing.” | Medical history of kidney stone disease in the questionnaire | High risk |
| F.Baowaidan, 2022 [71] | Retrospective | 190 | Median 32 months | 57.5 (21-93) | 115 (60.5) | Alcohol | Medical records | Renal colic and/or stone upon an imaging studies by CT or ultrasonography with radiography after endourological management | Some concerns |
| Parvin Khalili, 2021 [72] | Cross-sectional study | 9,932 | No | 49.94 ± 9.56 | 4,622 (46.5) | Alcohol, purified water | Self-administered questionnaire | Self-administered questionnaire | High risk |
| Fahimeh Haghighatdoost, 2021 [73] | Case-control study | 430 | NA | 39.4 ± 11.9 | 332 (77.2) | Potential renal acid load, net endogenous acid production | Self-reported, 147-item food frequency questionnaire | Pathologically confirmed by a nephrologist | Some concerns |
| Yaofei Sun, 2020 [74] | Population-based, retrospective cross-sectional study | 28,807 | No | 47.7 ± 16.8 | 13,975 (48.5) | Zinc | 24-hour dietary recall interview | Questionnaire "Have you ever had kidney stones?" and "How many times have you passed a kidney stone?" | High risk |
| Tahereh Azimi, 2020 [75] | Case-control | 634 | NA | 41.03 ± 11.03 (case), 38.00 ± 11.48 (control) | NA | Healthy diet pattern, unhealthy diet pattern | Food frequency questionnaire with 147 items of foods | Known case kidney stone patients who referred to the Hasheminejad and Moheb Hospitals in Tehran | High risk |

| | | | | | | | | | |
|---|---|---|---|---|---|---|---|---|---|
| Thomas J. Littlejohns, 2020 [76] | Prospective cohort | 439,072 | Mean 6.1 ± 0.97 year | NA | NA | Tea, coffee, water, alcohol, fruits, leafy vegetables, fiber, fish, oily fish, nonoily fish, meat, processed meat, poultry, red meat, cheese | Food-Frequency Questionnaire | Hospital inpatient records, ICD coding system | Some concerns |
| Yaofei Sun, 2020 [77] | Retrospective cross-sectional study | 16,115,050 | No | 55.89 | 9,270,183 (57.25 %) | Caffeine | Structured dietary recalls to determine caffeine intake | Questionnaire for recurrent kidney stones "Have you ever had kidney stones?" and " How many times have you passed a kidney stone?" | High risk |
| Xiang Shu, 2019 [78] | Prospective cohort | 127,220 | 8 years | NA | 58,054 (45.6) | Animal protein, dairy protein, nondairy protein, plant protein | Food-Frequency Questionnaire | Participants recalled the history of the urinary tract stone diagnosis and the date of the first diagnosis, the stone location and the imaging detection methods. | High risk |
| Hao Ping, 2019 [79] | Prospective cohort | 9,667 | Mean 33.5 (6-42) months | 46.2 | 7,310 (75.6) | Alcohol | Standardized questionnaire | Incident kidney stones confirmed via annual abdominal ultrasonography by certified urologists | High risk |
| Xiang Shu, 2019 [80] | Prospective cohort | 127,220 | NA | 52.4 ± 9.0 (women without | 58,054 (45.6) | Tea | Questionnaires | Self-reported by participants | High risk |

| | | | | | | | | | |
|---|---|---|---|---|---|---|---|---|---|
| | | | | kidney stone) 51.4 ± 8.3 (women with kidney stone) 55.3 ± 9.7 (men without kidney stone) 54.4 ± 9.1 (men with kidney stone) | | | | | |
| Hung-Yu Chen, 2018 [81] | Retrospective study | 13,842 | NA | 49.0 ± 12.9 (no stone), 51.8 ± 11.7 (stone) | 8,194 (59.2) | Tea, alcohol | Four closed-ended questions | Abdominal sonography by experienced radiologists who were unaware of the objectives of the study | Some concerns |
| WU Zhong Biao, 2017 [82] | Cross-sectional study | 8,807 | No | 42.25 ± 13.13 | 4,612 (52.4) | Tea | A standard questionnaire | Ultrasonography by well-trained examiners | High risk |
| Pietro Manuel Ferraro, 2017 [83]# | Prospective cohort | 192,126 | Median 11.5 (6.5, 16.7) years (HPFS), 12.1 (7.9, 20.0) years (NHS I), 11.3 (5.1, 16.0) | 54.2 ± 9.7 (HPFS), 52.9 ± 7.1 (NHS I), 36.6 ± 4.6 (NHS II) | 51,529 (26.8) | Water, DASH diet, calcium, sugar sweetened beverage | Food frequency questionnaire | Questionnaires (date of occurrence and accompanying symptoms) | Low risk |

| | | | | | | | | | |
|---|---|---|---|---|---|---|---|---|---|
| | | | years (NHS II) | | | | | | |
| YG Jiang, 2017 [84] | Cross-sectional study | 3,350 | No | 48.97 ± 17.02 | 1,091 (32.6) | White spirits | Questionnaire | Ultrasound examination | High risk |
| Pietro Manuel Ferraro, 2016 [85] | Prospective cohort | 193,676 | NA | 54.3 (9.8) (HPFS), 52.9 (7.1) (NHS I), 36.6 (4.6) (NHS II) | 51,529 (26.6) | Dairy protein, nondairy protein, potassium | Food frequency questionnaire | Questionnaires (date of occurrence and accompanying symptoms) | Low risk |
| Ai Zhao, 2015 [86] | Case-control study | 1,278 | No | 42.75 ± 11.87 | 455 (35.6) | Grain, vegetable, fruit, milk, meat, tea, alcohol, water | Interviewer-administered questionnaire, laboratory result | ICD-10 for diagnosed as having nephrolithiasis | High risk |
| Mathew D. Sorensen, 2014 [87] | Prospective cohort | 83,922 | Median 8 years | 64 ± 7 | 0 (0) | Fiber, fruit, vegetable | WHI food frequency questionnaire | Questionnaire | Some concerns |
| Pietro Manuel Ferraro, 2014 [88] | Prospective cohort | 217,883 | Mean 8.3 years (HPFS), 14 years (NHS I), 8.2 years (NHS II) | NA | 51,529 (23.6) | Caffeine | Food frequency questionnaire | Questionnaires (date of occurrence and accompanying symptoms) | Low risk |
| Abdul Haleem Panhwar, 2013 [89] | Cross-sectional study | 145 | No | NA | 108 (75) | Underground water, bottled mineral water, and domestic treated water | Questionnaire survey | X-ray and ultrasound reports | High risk |
| Eric N. Taylor, 2013 [90] | Prospective study | 226,627 | 20 years (HPFS), 20 years | 48.7 (HPFS), 52.5 (NHS I), 36.1 (NHS II) | 30762 (100) (HPFS) | Non-dairy dietary calcium, dairy calcium | Food frequency questionnaires every 4 years | Biennial mailed questionnaires inquire on newly | Low risk |

| | | | | | | | | | |
|---|---|---|---|---|---|---|---|---|---|
| | | | (NHS I), 16 years (NHS II) | | , 0 (0) (NHS I and NHS II) | | | diagnosed disease such as kidney stones | |
| A Trinchieri, 2013 [91] | Case-control study | 246 | NA | 40.8 ± 13.2 (control), 41.4 ± 12.6 (case) | 120 (48.8) | Potential renal acid load | Dietary histories by 24 hours recall | Infrared spectrometry | High risk |
| Pietro Manuel Ferraro, 2013 [92] | Prospective cohort | 194,095 | NA | 54 (10) (HPFS), 51 (7) (NHS I), 37 (5) (NHS II) | 51,529 (26.5) | Sugar-sweetened cola, artificially sweetened cola, sugar-sweetened noncola, artificially sweetened noncola, coffee, decaffeinated coffee, tea, red wine, white wine, beer, liquor, apple juice, grapefruit juice, orange juice, tomato juice, other fruit juice, punch, whole milk, skim milk, water | Food frequency questionnaire | Questionnaires (date of occurrence and accompanying symptoms) | Low risk |
| Meng Dai, 2013 [93] | Case-control study | 2,006 | NA | NA | 1,290 (64.3) | Grain, root stock, tubers, leafy vegetables, bean products, fruit, egg, dairy product, meat and fish, oil, nuts, Guangzhou dessert, Guangzhou soup, fluid intake | Brief food frequency questionnaire derived from the China National Nutrition and Health Survey 2002 | ICD-10: N20.0, N13.201, B-ultrasonic image | High risk |

| | | | | | | | | | |
|---|---|---|---|---|---|---|---|---|---|
| Jie Tang, 2012 [94] | Cross-sectional study | 15,444 | No | 43.5 ± 24.3 | 7,197 (46.6) | Protein, water, zinc | 24 hour recalls of food and beverage intake data | Standardized home interview self-report question: "Have you ever had a kidney stone?". | High risk |
| Mathew D. Sorensen, 2012 [95] | Prospective cohort | 78,293 | Mean 8 years | NA | 0 (0) | Water, sodium, calcium | Validated Women's Health Initiative Observational Study (WHI) food frequency questionnaire | Self-reported annually through year 8 | Some concerns |
| Eric N. Taylor, 2009 [96] | Prospective cohort | 241,766 | 18 years (HPFS), 18 years (NHS I), 14 years (NHS II) | NA | 45821 (100) (HPFS), 0 (0) (NHS I and NHS II) | DASH diet | Semiquantitative food frequency questionnaire | Questionnaires about newly diagnosed kidney stones | Low risk |
| Eric N. Taylor, 2007 [97] | Prospective study | 240,681 | 16 years (HPFS), 18 years (NHS I), 10 years (NHS II) | NA | 45,985 (100) (HPFS), 0 (0) (NHS I and NHS II) | Oxalate | Food frequency questionnaire | The biennial questionnaire with medical records | Some concerns |
| Eric N. Taylor, 2005 [98] | Prospective study | 234,786 | 14 years (HPFS), 14 years (NHS I), | NA | 46,403 (100) (HPFS), 0 (0) NHS I | Arachidonic acid, linoleic acid, EPA+DHA | Food frequency questionnaire | Subjects reported on the interval diagnosis of kidney stones every 2 years with confirmation by | Low risk |

| | | | | | | | | | |
|---|---|---|---|---|---|---|---|---|---|
| | | | 8 years (NHS II) | | and NHS II) | | | medical records from random sample | |
| Eric N. Taylor, 2004 [99] | Prospective cohort | 45,619 | 14 years | 45 | 45,619 (100) | Animal protein, water, calcium | Semiquantitative food frequency questionnaire | Patients reported on the interval diagnosis of kidney stones every 2 years | Low risk |
| Gary C. Curhan, 2004 [21] | Prospective cohort | 96,245 | NA | NA | 0 (0) | Calcium, animal protein, sucrose, phytate, fluid | Validated semi-quantitative Food Frequency Questionnaires | Self-reported diagnosis | Low risk |
| Ying-Huei Lee, 2002 [100] | Case-control | 4,588 | NA | 47.8 ± 13.4 (male) 43.2±11.9 (female) | 2,378 (51.8) | Alcohol, water, tea | Self-administered questionnaire | Self-reported history of upper urinary calculi | High risk |
| W. Dallas Hall, 2001 [101] | Case-control study | 2,358 | NA | 63.5 ± 7.1 (case), 63.5 ± 7.1 (control) | 0 (0) | Alcohol, calcium, sodium, potassium, magnesium, protein, animal protein, oxalic acid, sucrose | Standardized questionnaire | Questionnaire to a history of kidney or bladder stones | High risk |
| Yu-Ying Chen, 2000 [102] | Longitudinal cohort study | 7,784 | Mean 3.3 ± 2.8 years | NA | NA | Water hardness | Summary report from the National Stream Quality Accounting Network of the United States Geological Survey | Abnormal concretion in the kidney or ureter documented by x-ray evidence of its location | High risk |
| Gary C. Curhan, 1998 [103] | Prospective cohort | 81,093 | 8 | NA | 0 (0) | Water, skim milk, whole milk, apple juice, orange juice, grapefruit juice, tomato juice, other fruit juice, punch/lemonade, soda, | Semiquantitative food frequency questionnaires | Questionnaire ask whether they had ever had a kidney stone diagnosed and the date of first occurrence | Low risk |

| | | | | | | | | | |
|---|---|---|---|---|---|---|---|---|---|
| | | | | | | coffee, tea, beer, wine, liquor | | | |
| Gary C. Curhan, 1997 [24] | Prospective cohort | 91,731 | 12 years | NA | 0 (0) | Calcium, animal protein, sodium, potassium, sucrose, water | Semiquantitative food frequency questionnaires | Questionnaire ask whether the women had ever received a diagnosis of a kidney stone and if so, the date of the first occurrence | Low risk |
| Gary C. Curhan, 1996 [104] | Prospective study | 45,289 | 6 years | NA | 45,289 (100) | Water, skim/low fat milk, whole milk, other fruit juice, apple juice, orange juice, grapefruit juice, tomato juice, punch/lemonade, cola with caffeine, cola without caffeine, noncola, coffee, tea, beer, wine, liquor | Semiquantitative food frequency questionnaire | Self-reported questionnaire | Low risk |
| Gary C. Curhan, 1993 [23] | Prospective study | 45,619 | NA | NA | 45,619 (100) | Calcium, animal protein, potassium, fluid | Semiquantitative food frequency questionnaire | Questionnaires | Low risk |

Reference numbers correspond to the reference list in the main manuscript

## Supplementary Figure S1 Study Workflow

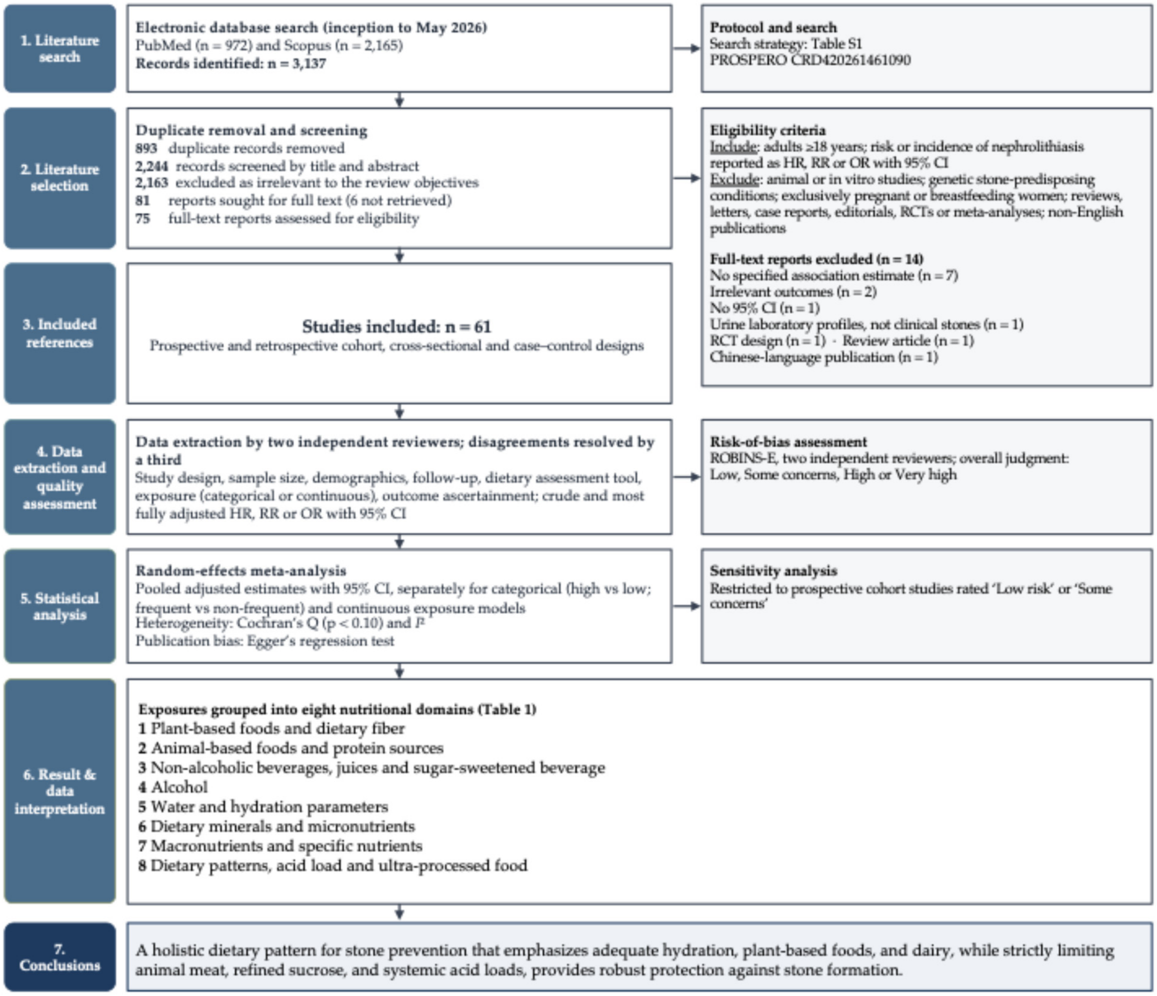

Supplement: Supplementary file 1 [file nutrients-18-03653-s001.zip › nutrients-4601672-supplementary.pdf]
